# The molecular pathophysiology of chronic non-bacterial osteomyelitis (CNO)—a systematic review

**DOI:** 10.1186/s40348-017-0073-y

**Published:** 2017-07-06

**Authors:** Sigrun Ruth Hofmann, Franz Kapplusch, Katrin Mäbert, Christian Michael Hedrich

**Affiliations:** 1 Pediatric Rheumatology and Immunology, Children’s Hospital Dresden, Faculty of Medicine Carl Gustav Carus, Technische Universität Dresden, Fetscherstr. 74, D-01307 Dresden, Germany; 2 Department of Women’s & Children’s Health, Institute of Translational Medicine, University of Liverpool, Liverpool, UK; 3 Department of Paediatric Rheumatology, Alder Hey Children’s NHS Foundation Trust Hospital, Liverpool, UK

**Keywords:** Chronic non-bacterial osteomyelitis, CNO, Chronic recurrent multifocal osteomyelitis, CRMO, Inflammation, Cytokine, Mechanism

## Abstract

Chronic non-bacterial osteomyelitis (CNO) belongs to the growing spectrum of autoinflammatory diseases and primarily affects the skeletal system. Peak onset ranges between 7 and 12 years of age. The clinical spectrum of CNO covers sometimes asymptomatic inflammation of single bones at the one end and chronically active or recurrent multifocal osteitis at the other.

Despite the intense scientific efforts, the exact molecular mechanisms of CNO remain unknown. Recent data suggest CNO as a genetically complex disorder with dysregulated TLR4/MAPK/inflammasome signaling cascades resulting in an imbalance between pro- and anti-inflammatory cytokine expression, leading to osteoclast activation and osteolytic lesions.

In this manuscript, the current understanding of molecular patho-mechanisms in CNO will be discussed.

## Introduction

Chronic non-bacterial osteomyelitis (CNO) is an autoinflammatory bone disorder. It primarily affects children and adolescents with a peak onset between 7 and 12 years. However, CNO can generally occur in all age groups. The clinical spectrum is variable and ranges from single asymptomatic bone lesions up to the most severe form of chronic recurrent multifocal osteomyelitis (CRMO) [1–7]. Due to this variable clinical presentation with sometimes rather mild and unspecific clinical symptoms, diagnosis is frequently delayed or even missed. This is particularly worrying, since untreated CNO may result in bone sclerosis, pathological fractures (mainly of the vertebral bodies) sometimes with subsequent neurological symptoms, growth anomalies, pain amplification, and psychosocial problems [8–11].

Recent discoveries contributed to a better understanding of underlying molecular mechanism leading to systemic inflammation in CNO. However, the exact molecular pathophysiology remains incompletely understood [9].

## Cytokine dysregulation in CNO/CRMO

To our current understanding, several molecular disturbances contribute to the molecular pathophysiology of CNO. Bone inflammation may be the result of dysbalanced cytokine expression from innate immune cells and subsequent osteoclast differentiation and activation, resulting in bone remodeling and inflammatory bone loss [4–6, 9, 12, 13].

### Dysbalanced cytokine expression

**Fig. 1:**
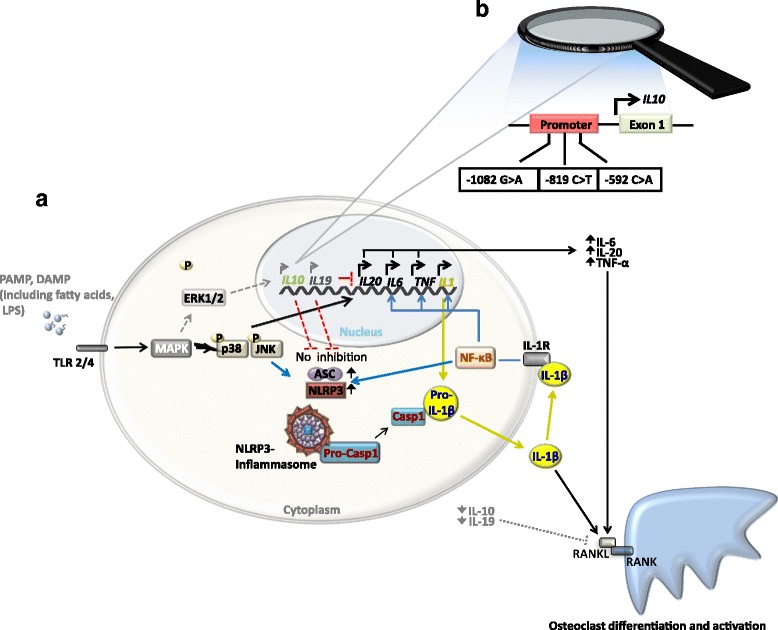
Molecular pathophysiology of CRMO. **a** The sensing of danger- and pathogen-associated molecular patterns (DAMPS and PAMPS) occurs by pattern recognition receptors (PRRs), such as membrane-associated Toll-like receptors (TLRs) and cytoplasmic-localized NOD-like receptors (NLRs). TLR4 activation by lipopolysaccharide (LPS) results in the activation of mitogen-activated protein kinases (MAPK), including extracellular signal-regulated kinases 1 and 2 (ERK1/2), p38, and Jun kinase (JNK). In response to the recognition of danger signals, multiprotein complexes, so-called inflammasomes, are activated. The NLRP3 inflammasome comprises NLRP3, ASC, and procaspase-1. Inflammasomes mediate caspase-1 activation, which results in cleavage of pro-IL-1β into its active from IL-1β. In monocytes from CRMO patients, impaired ERK1/2 signaling results in failure to express IL-10 and IL-19. Reduced expression of IL-10 and IL-19 contributes to inflammasome activation and IL-1β release. Pro-inflammatory cytokines IL-1β, IL-6, IL-20, and TNFα increase the interaction of RANK receptors on osteoclast precursor cells with their soluble ligand RANKL, inducing osteoclast activation and differentiation. **b** *IL10* promoter polymorphisms rs1800896 (-1082A>G), rs1800871 (-819T>C), and rs1800872 (-592A>C) form distinct haplotypes (GCC, ACC, and ATA) that determine transcription factor recruitment. (MAPK mitogen-activated protein kinase; CRMO chronic recurrent multifocal osteomyelitis; ERK1/2 extracellular signal-regulated kinases 1 and 2; TLR Toll-like receptor; IL interleukin; JNK Jun kinase; TNF tumor necrosis factor; NF-κB nuclear factor-κB; Casp1 caspase-1; PAMP pathogen-associated molecular pattern; DAMP danger-associated molecular pattern; RANK receptor activator of nuclear factor-κB; RANKL RANK ligand)

At the time of diagnosis, treatment naïve patients with CRMO (the most severe form of CNO) exhibit increased serum levels of pro-inflammatory cytokines IL-6 and TNF-α, while the immune-regulatory cytokine IL-10 was not detectable [14, 15]. This observation, together with the assumption that CNO/CRMO may be an autoinflammatory disorder, led to scientific efforts targeting cytokine expression from innate immune cells, namely monocytes. In response to stimulation of Toll-like receptor 4 (TLR4) with lipopolysaccharide (LPS), monocytes from CRMO patients failed to produce IL-10, while expressing increased amounts of IL-6 and TNF-α [15]. Interleukin-10 is eponymous to the IL-10 cytokine family, which (among others) includes the homologues IL-19 (also immune-regulatory) and IL-20 (pro-inflammatory) [16, 17]. Together with *IL10*, the *IL19* and *IL20* genes are organized in the same so-called *IL10* cytokine cluster on chromosome 1q32. In monocytes from CRMO patients, expression of IL-19 is decreased when compared to controls, while expression of the pro-inflammatory cytokine IL-20 is enhanced in response to stimulation with LPS (Fig. 1) [15, 18].

Impaired IL-10 expression in monocytes from CRMO patients is (at least partially) caused by reduced activation of mitogen-activated protein kinases (MAPK) extracellular signal-regulated kinase (ERK)1 and ERK2 (Fig. 1) [18]. Attenuated ERK activation resulted in impaired phosphorylation and nuclear shuttling of the transcription factor signaling protein (Sp)-1, subsequently leading to reduced recruitment of Sp-1 to regulatory elements at the *IL10* and *IL19* promoters. Expression of pro-inflammatory cytokines, including IL-6 and TNFα, though also depending on MAPK activation, is increased in monocytes from CRMO patients. This, however, may be due to unaffected activation of alternative MAPK pathways, such as p38 MAPK (Fig. 1) [12, 15, 18].

Post-translational modifications to the amino-terminus of histone proteins and the addition of methyl groups to the 5′-carbon end of cytidine residues in cytidine-phosphate-guanosine sequences of the genomic DNA (DNA methylation) represent the two main mechanisms during chromatin remodeling [19–21]. MAP kinases, including ERK1 and ERK2, are responsible for the phosphorylation of histone protein amino termini. Impaired activation of ERK leads to reduced histone 3 serine 10 (H3S10) phosphorylation at the *IL10* and *IL19* promoters in monocytes from patients with CRMO (Fig. 1) [15, 18]. Histone H3S10 phosphorylation is an activating epigenetic modification, resulting in an “opening” of regulatory regions to transcription factor binding and RNA polymerase recruitment [19, 20]. Therefore, reduced H3S10 phosphorylation further contributes to reduced Sp-1 recruitment. Also, DNA methylation is altered in monocytes from CRMO patients. DNA methylation patterns in CRMO are complex and incompletely understood. Increased DNA methylation (an inactivating epigenetic modification) of *IL19* contributes to impaired IL-19 expression, while methylation of the proximal *IL20* promoter was reduced, contributing to increased IL-20 expression [6, 9, 12, 15, 18, 22].

### Osteoclast activation and differentiation

We demonstrated that monocytes from CRMO patients express increased levels of pro-inflammatory cytokines (IL-6, TNF-α, IL-20), while they fail to produce immune-regulatory cytokines IL-10 and IL-19 [18, 22].

The question of how imbalanced cytokine expression may translate into the bone inflammation and the expression of CRMO remained unanswered. Recently, NLRP3 inflammasome activation was linked to inflammatory bone loss and synovial inflammation in IL-10-deficient mice [23]. Two studies demonstrated increased NLRP3 and IL-1 mRNA expression in peripheral blood mononuclear cells (PBMCS) [24] and monocytes [22] of CRMO patients. We linked impaired IL-10 and IL-19 expression with subsequently increased IL-1β mRNA expression and IL-1β proteins in monocytes from CRMO patients [22]. Indeed, enhanced inflammasome activation and IL-1β secretion from monocytes was reversed by co-culture with recombinant IL-10 or IL-19 [22].

Taken together, observations in IL-10-deficient animals and CRMO patients support the hypothesis that imbalanced expression of anti-inflammatory (IL-10, IL-19) and pro-inflammatory (IL-1β, IL-6, TNFα, IL-20) cytokines result in bone inflammation [6, 22]. Data from metabolic studies indicate that imbalanced expression of anti- and pro-inflammatory cytokines contribute to enhanced interactions between the receptor activator of nuclear factor-κB (RANK) surface receptor and its soluble ligand RANKL on osteoclast precursor cells (Fig. 1) [13, 25, 26]. Interactions between RANK and RANKL result in osteoclast differentiation and activation and may therefore contribute to inflammatory bone loss in CNO. However, the involvement of these molecular events in CNO remains to be scientifically proven.

## CNO as a genetically complex disorder

Genetic predisposition for CNO/CRMO has been suspected by the occurrence of familial monogenic disorders including non-infectious osteomyelitis as one of the main clinical features. Patients with Majeed syndrome (caused by homozygous mutations in the *LPIN2* gene) [27], the deficiency of interleukin-1 receptor antagonist (DIRA; caused by autosomal recessive loss-of-function mutations in *IL1RN*) [28–30], and pyogenic arthritis, pyoderma gangrenosum and acne syndrome (PAPA; caused by autosomal dominant loss-of-function mutations in *PSTPIP1*) [31] develop severe aseptic osteomyelitis. Because of the central involvement of increased IL-1 signaling and inflammasome activation in these monogenic disorders involving CNO as a key symptom, inflammasome activation and IL-1 release appeared likely involved in the pathophysiology of “sporadic” CNO. However, screening of *IL1RN* did not deliver disease-causing mutations in a cohort of CNO patients [32].

Associations of CNO/CRMO with other autoinflammatory disorders, such as psoriasis or inflammatory bowel disease, and the occurrence of familial clusters further suggest a common genetic predisposition for sporadic CNO [1, 7, 33, 34]. Golla et al. performed an association study in CNO patients identifying a potential susceptibility locus on chromosome 18q21.3-22 [35]. However, findings of this study were not confirmed in subsequent efforts in larger and independent cohorts. Recently, we analyzed the polymorphisms in the proximal promoter region of the immune-regulatory cytokine interleukin 10 (*IL10*) in a cohort of CRMO patients. Three *IL10* promoter polymorphisms rs1800896 (c.-1082A>G), rs1800871 (c.-819T>C), and rs1800872 (c.-592A>C) form distinct haplotype blocks (GCC, ACC, and ATA) that influence transcription factor recruitment capacities to regulatory elements within the proximal promoter [16, 17]. Based on promoter haplotypes, IL-10 expression can be stratified from “high” (GCC), over “medium” (ACC), to “low” (ATA). Previously, recruitment of Sp-1 was shown to depend on “permissive” GCC *IL10* promoter haplotypes, which may contribute to disease outcomes in infectious disorders [15, 17, 36]. Unexpectedly, we determined an enrichment of *IL10* promoter haplotypes encoding for “high” IL-10 expression (GCC), while we did not find a single CNO patient with homozygous ATA haplotypes in our cohort. Thus, *IL10* polymorphisms can by themselves certainly not explain impaired IL-10 secretion by monocytes from CRMO patients [15, 17]. Therefore, it appeared likely that additional molecular mechanisms contribute to altered IL-10 activation and secretion in CNO (see above). Indeed, the absence of *IL10* promoter haplotypes encoding for “low” IL-10 expression suggests that individuals with the aforementioned molecular defects contributing to reduced IL-10 expression in CNO/CRMO and ATA haplotypes may develop more severe symptoms and will therefore not be diagnosed with CNO but potentially some other inflammatory condition. While this hypothesis sounds promising, it currently remains to be investigated and proven in larger multi-national cohorts.

Recently Cox et al. identified mutations in *FBLIM1* in two CNO patients from South Asia using whole-exome sequencing [37]. While the exact function of Filamin Binding LIM Protein 1 (FBLIM1) remains somewhat unclear, it acts as an anti-inflammatory molecule by regulating receptor activator of NF-κB ligand (RANKL) activation [38]. Interactions between RANK receptors on the surface of osteoclast precursor cells and soluble RANKL result in osteoclast generation and activation, thereby contributing to bone remodeling and inflammatory bone loss [13, 25, 26]. Expression of *FBLIM1* is regulated by STAT3 [37]. Since IL-10 induces the activation of STAT3, reduced IL-10 expression may result in impaired STAT3 activation and subsequently altered *FBLIM1* expression. Indeed, both reported patients carry *IL10* promoter haplotypes encoding for “low” IL-10 expression. Therefore, the combination of *IL10* promoter haplotypes encoding for “low” gene expression together with *FBLIM1* variants may result in CNO [16, 39].

## Therapeutic implications

Treatment of CNO is based on personal experience, expert opinion, case reports, and small case series. Nonsteroidal anti-inflammatory drugs (NSAIDs) are generally used as first-line therapy [11, 14, 40–45]. Anti-inflammatory effects of NSAIDs are achieved through inhibition of cyclooxygenase enzymes. Recently, NSAIDs were demonstrated to exert variable suppressive effects on inflammasomes [46]. Efficacy of NSAIDs in CNO may therefore be explained by the involvement of pro-inflammatory monocytes in the pathophysiology of CNO that likely contribute to the generation and activation of osteoclasts. Prostaglandins are involved in osteoclast activation, and the inflammasome activation is a hallmark of pro-inflammatory monocytes in CNO [6]. As NSAIDs, corticosteroids suppress prostaglandin production, which is achieved through the inhibition of phospholipase A1. Furthermore, corticosteroids reduce the expression of NFκB-mediated pro-inflammatory cytokines, including IL-1, IL-6, and TNF-α [6]. Some authors report successful short-term use of corticosteroids in CNO, usually over a few weeks [6, 40].

Tumor necrosis factor α centrally contributes to bone inflammation in CNO. Thus, blockade of TNF-α appears a promising therapeutic intervention. Indeed, several small case series report induction of clinical and radiological remission in CNO in response to TNF blockade [6, 8, 11, 40, 47, 48].

The bisphosphonate pamidronate inhibits osteoclast activity and exerts incompletely understood effects on inflammatory cytokine expression [6]. Pamidronate was reported to induce rapid and long-lasting remission in most CNO patients [6, 8, 49–52].

Provided the previously described overactivation of inflammasomes and increased IL-1 release from PBMCs and monocytes from CNO patients, surprisingly, few cases of anti-IL-1 treatment have been reported with mixed response and variable outcomes (Girschick H.J. et al., unpublished data). The potential explanation for the poor response may include low tissue concentrations and pathophysiological heterogeneity in CNO among others.

## Conclusions

Dysregulated cytokine expression from monocytes centrally contributes to the inflammatory phenotype of CNO. Recent reports on genetic and molecular alterations in CNO patients indicate complex and variable immune pathology. Response to previously empiric treatment can now be explained, based on molecular patterns in immune cells from CNO patients.
